# First Death in P. M. B. A.

**Published:** 1885-12

**Authors:** 


### THE FIRST DEATH IN THE P. M. B. A. OF TEXAS.

Although this Association was organized in April, 1884, there has not occurred a death amongst its members until November 4, 1885. Brother W. H. Park, an eminent physician and surgeon, of Tyler, Texas, died on that day of dengue, or the effects of dengue. Assessments of $1 each were immediately sent out to the surviving members for the benefit of Mrs. Park, and, at this writing (December 10th), nearly all have responded. There are at present ninety members in good standing, but as those who joined since November 4th are not liable to this assessment, the total amount collected will not exceed $60. The constitution allows thirty days in which to pay any assessment.

The time for payment will expire on the 13th inst., when those who have not responded will forfeit their membership. In our next we hope to publish the list and the amount realized from the assessment, and the widow's receipt for the money. Though small, the amount will, we hope, be acceptable and timely.

The P. M. B. A. will become a feature of the Texas profession, and we hope to see much good result from it. Whatever the amount of money may be, that is realized from an assessment, large or small, it cannot be felt by the members, while the aggregate may be a God-send to the recipients; it may save the sacrifice of property, or save embarrassments, to say the least; and, coming as a free-will offering from a brotherhood of physicians composed of the better element of the profession, it would have a double value, and none of the aspects of charity. We hope to see our State Association take hold of the matter. Why should there not be passed, at next meeting, a resolution that, in case of the death of a member, each would contribute $1 for a fund for the widow or other dependencies? That would accomplish the object. The sum then would be a very considerable life insurance, and it would add another inducement to become a member, and to keep up one's membership by payment of dues. We learn some sixty members were dropped for non-payment of dues this year.

This Association has had a hard struggle for recognition; but not discouraged by the cold water thrown on it, the utter indifference of the profession to our appeals in the name of humanity, we have kept pegging away, until now it is fairly on its legs, and growing like "a two-year old." The day will come when the profession will be proud of it.

